# Identity Functioning in Patients with an Eating Disorder: Developmental Trajectories throughout Treatment

**DOI:** 10.3390/nu16050591

**Published:** 2024-02-21

**Authors:** Margaux Verschueren, Laurence Claes, Nina Palmeroni, Leni Raemen, Philip Moons, Liesbeth Bruckers, Geert Molenberghs, Eva Dierckx, Katrien Schoevaerts, Koen Luyckx

**Affiliations:** 1 De Korbeel, Child and Adolescent Psychiatric Clinic, 8500 Kortrijk, Belgium; margaux.verschueren@gmail.com; 2 Faculty of Psychology and Educational Sciences, KU Leuven, 3000 Leuven, Belgium; leni.raemen@kuleuven.be (L.R.); koen.luyckx@kuleuven.be (K.L.); 3 Child & Youth Institute, KU Leuven, 3000 Leuven, Belgium; 4 Faculty of Medicine and Health Sciences, University of Antwerp, 2610 Wilrijk, Belgium; 5 Universitair Psychiatrisch Centrum KU Leuven, 3070 Kortenberg, Belgium; nina.palmeroni@upckuleuven.be; 6 Department of Public Health and Primary Care, KU Leuven, 3000 Leuven, Belgium; philip.moons@kuleuven.be; 7 Institute of Health and Care Sciences, University of Gothenburg, 40530 Gothenburg, Sweden; 8 Department of Paediatrics and Child Health, University of Cape Town, Cape Town 7700, South Africa; 9 I-BioStat, Data Science Institute, Hasselt University, 3500 Hasselt, Belgium; liesbeth.bruckers@uhasselt.be (L.B.); geert.molenberghs@kuleuven.be (G.M.); 10 I-BioStat, KU Leuven, 3000 Leuven, Belgium; 11 Psychiatric Hospital Alexianen Zorggroep Tienen, 3300 Tienen, Belgium; eva.dierckx@vub.be (E.D.); katrien.schoevaerts@azt.broedersvanliefde.be (K.S.); 12 Faculty of Psychology and Educational Sciences, Vrije Universiteit Brussel, 1050 Brussels, Belgium; 13 Educational Unit for Professional Training and Service in the Behavioural Sciences (UNIBS), University of the Free State, Bloemfontein 9301, South Africa

**Keywords:** identity, synthesis, identity confusion, eating disorder symptomatology, treatment, latent trajectories

## Abstract

Increasing research has indicated a strong association between identity functioning and eating disorder (ED) symptomatology. However, a detailed investigation of identity throughout ED treatment is lacking. The present longitudinal study examined identity in inpatients with an ED and explored its simultaneous change with ED symptomatology throughout treatment. A total of 225 female patients completed questionnaires at admission. From these 225 patients participating at admission (Wave 1), 110 also participated in at least one additional measurement wave, with 43.64% (*n* = 48) participating at admission and during treatment, 16.36% (*n* = 18) participating at admission and at discharge, and 40% (*n* = 44) participating at admission, during treatment and at discharge. Questionnaires on identity synthesis, identity confusion, identity processes, and ED symptomatology were completed. Latent growth curve modeling was used to address the research questions. Throughout treatment, a decrease in identity confusion and an increase in identity synthesis and adaptive identity processes were found. Accordingly, increases in identity synthesis and identification with commitment were related to general decreases in the drive for thinness and body dissatisfaction. Similarly, such decreases in ED symptoms were related to general decreases in identity confusion and ruminative exploration. The present study points to an increase in identity functioning throughout treatment, and longitudinal associations between identity functioning and ED symptomatology were found. Helping patients to decrease their ruminative exploration and to increase their identification with previously made life commitments and treating body/weight concerns could both be helpful in ED treatment.

## 1. Introduction

To understand the development of eating disorders (EDs), research increasingly focuses on identity as an important factor [1,2,3]. EDs often develop in adolescence [4,5], a crucial period that challenges individuals to figure out who they are or who they want to become in life [6]. Research indicates that adolescents who struggle with this identity task, are at risk of experiencing ED symptomatology over time [2]. Similarly, patients with an ED experience severe identity issues when compared to community controls, e.g., [7,8,9]. It has been recommended to promote a healthy identity development in ED prevention programs, as bolstering one’s identity may have long-term, positive changes in ED symptomatology [10]. Hence, the present study focused on identity functioning in patients with an ED and investigated the simultaneous change of identity functioning and ED symptomatology throughout ED treatment.

### 1.1. The Process of Identity Formation

Adolescence and the transition to adulthood are key developmental periods in which individuals are faced with important identity questions [6]. Such identity questions are challenging to youth, often resulting in identity confusion (feelings of being mixed up and lacking a clear purpose in life) [6]. Only when a coherent sense of self is experienced that supports self-directed decision making, identity synthesis is reached [6,11]. Extending Marcia’s [12] work, Luyckx et al. [13] developed a process-oriented identity model, integrating both adaptive and maladaptive identity processes as behavioral markers of identity synthesis and confusion. When adolescents’ identity develops smoothly, individuals often start by actively exploring different alternatives (exploration in breadth), which allows them to make decisions about important issues in life (commitment making). This identity choice can be re-evaluated on whether it truly corresponds with internal values and aspirations (exploration in depth) and can become integrated into one’s sense of self (identification with commitment). Finally, Luyckx et al. [13] describe the maladaptive process of ruminative exploration, which is characterized by continuously worrying and feeling stuck in ongoing exploration. To grasp identity formation, it is important to assess identity in terms of both the degree of synthesis or confusion as well as the behavioral identity processes. Such an integrative perspective, however, is largely lacking in patients with an ED.

### 1.2. Identity and ED Symptomatology

In ED theories, identity disturbance has been presented as one of the main issues in ED pathology. The transdiagnostic theory of EDs [14] claims that a dysfunctional system of self-evaluation is the core psychopathology in most EDs (such as anorexia nervosa (AN), bulimia nervosa (BN), and binge eating disorder (BED)). Patients with an ED judge themselves almost exclusively based on (their ability to control) their body weight/shape, whereas alternative sources of self-worth are lacking. This overvaluation of body size/weight causes these patients to experience a narrowly defined identity [10,15]. Additionally, Bruch [16,17] argued that a lack of a clear identity can play a role in the development of AN, as the body—being highly controllable and culturally valued—could represent a (maladaptive) source of self-definition in these individuals [7,15].

In recent years, researchers have studied identity formation in relation to ED symptomatology and have recognized their close relation in community and clinical samples [1,2,18]. Verschueren, Luyckx, et al. [9] found that female patients with an ED experienced substantially more identity difficulties as compared to age-matched community controls. Patients scored lower on commitment making, identification with commitment, and exploration in breadth, and scored higher on ruminative exploration. However, the cross-sectional design of this study does not allow the tracing of the development of identity and the investigation of its simultaneous change with ED symptomatology during treatment. Moreover, while research in community samples has demonstrated that identity functioning is a dynamic and lifelong process [6,19,20,21], longitudinal research in clinical ED samples is necessary to investigate whether identity dimensions are malleable in ED patients as well.

Interestingly, identity issues do not only seem to increase ED symptomatology, but ED symptomatology may also hinder identity development. A longitudinal study in community adolescents [2] has pointed to bidirectional relations between identity and ED symptoms over time. While identity confusion increased vulnerability to ED symptomatology and identity synthesis protected against its development over time, ED symptomatology also positively predicted identity confusion and negatively predicted identity synthesis. Various theorists have indeed described how binge eating and purging can function as ways to escape from self-awareness [3,7]. Doing so, these patients do not actively engage in identity work, which only reinforces their initial sense of identity confusion [18].

### 1.3. ED Treatment and Outcome

Previous research has pointed to the large variability in ED treatment response [22,23]. A meta-analysis [24] concluded that patients who, at admission, present with lower ED severity, less psychopathology, or a greater motivation to recover, generally present with a better response at the end of treatment. Additionally, Sansfaçon et al. [25] found that patients who experience an intrinsic drive to change their ED behaviors and who were personally engaged (i.e., autonomous motivation) at the start of treatment presented with a stronger decrease in ED symptomatology throughout treatment.

In their meta-analysis, Vall and Wade [24] also described early symptom improvement as an important process in the prediction of treatment outcome, as patients who experience symptom reduction early in treatment, generally acquire a better final treatment outcome. In patients with BN [26], such rapid responders have been found to present with higher levels of self-directedness—an identification with personal goals, self-determination, and accepting responsibility of one’s choices [27]. More recent studies have also differentiated rapid, slow, and non-responders in patients with an ED [28,29], and found that ED severity (i.e., symptom frequencies, BMI, weight-related self-evaluation) and psychological/demographic variables (i.e., anxiety, depression, age, chronicity) could not predict treatment course. However, as rapid responders were able to make immediate symptom changes, the authors propose that these patients may be less entrenched in their ED [28]. Taken together, these findings emphasize that self-related constructs may play an important role in ED symptom reduction—emphasizing the need to explicitly attend to associations linking ED treatment response to identity functioning.

### 1.4. The Present Study

The present study was guided by three main objectives. First, we investigated whether identity functioning would differ among age groups (adolescent/adult), ED subtypes (restrictive/binge–purging), and previous admissions at an ED treatment center (yes/no). We hypothesized that patients with a chronic ED would be more vulnerable for identity issues [9]. ED patients often experience diagnostic shifts in ED development, going from a more restrictive subtype in early adolescence to a binge eating–purging subtype in late adolescence or adulthood [30,31,32]. Hence, we expected worse identity functioning in adult patients, patients with an ED of the binge eating–purging subtype, and patients with previous admissions at the treatment unit, as they are all more likely to experience chronic ED psychopathology.

Second, we examined identity development throughout an ED treatment. During treatment, patients are challenged to work on various factors that play a role in the onset and maintenance of their ED, in which they also learn to identify themselves as separate from their ED. Reducing ED symptoms can confront them with (previously disguised) feelings of identity confusion [15], allowing them to explore (new) identity options and make life commitments. Hence, we expected patients to increase in identity synthesis and the adaptive identity processes and to decrease in identity confusion and ruminative exploration throughout treatment.

Third, we investigated whether ED symptomatology would change simultaneously with identity functioning throughout treatment. We expected that improvements in ED symptoms would be related to an increase in identity functioning as well. Following previous longitudinal research in community adolescents [2], we expected identity synthesis to be negatively related over time to drive for thinness, body dissatisfaction, and bulimia, whereas identity confusion would be positively related to body dissatisfaction and bulimia. With regard to the identity processes, our hypotheses were less specific as no previous study has focused on their longitudinal interplay with ED symptoms. We tentatively expected that improvements in ED symptoms would be related to an increase in the adaptive identity processes and a decrease in ruminative exploration.

## 2. Materials and Methods

### 2.1. Procedure

Data were obtained from inpatients admitted to a specialized female ED treatment center in Tienen (Belgium) that adopts a directive therapy approach. First, at admission, patients are placed in a motivation orientation group (lasting 3 to 5 weeks) which challenges them to actively reflect upon their eating behaviors and on whether they are motivated to start intensive treatment. If so, they start a multidisciplinary treatment program. At the start of this program, patients filled out an informed consent form before participation and minors also provided informed consent from their parents. Participating patients then filled out the questionnaires of the present study (Wave 1). Information on diagnosis, treatment duration, illness duration, BMI, and previous admissions at the ED treatment center were retrieved from clinical records. If patients were admitted several times throughout the study period, only the data from the first admission were used in the present study. Patients’ diagnoses were established by (1) clinical interviews carried out by experienced psychiatrists/psychologists, (2) patient scores on the Eating Disorder Evaluation Scale (EDES) [33], and (3) patients’ BMI. Depending on their age (adolescent/adult) and BMI (below/over 16), patients are placed in different treatment groups. BMI < 16-treatment predominantly focuses on symptom reduction and weight gain, whereas BMI > 16-treatment also targets trauma and concurrent personality pathology or other co-occurring disorders. All patients participate in group/family/individual psychotherapy, socio-therapy, psychomotor and expressive therapy, and psychoeducation. After approximately 12 weeks of the treatment program, patients were asked again to fill out questionnaires (Wave 2), as this time frame (between 5 and 17 weeks of admission) is typically characterized by the greatest symptom reduction. However, at this time, some patients have already been discharged, some patients then start the discharge phase of approximately 4 weeks, whereas other patients (often with BMI < 16 at admission) still need more therapy. At the start of the discharge phase, patients were asked again to fill out questionnaires (Wave 3). Generally, admission duration varies between 4 and 6 months and discharge is related to an evaluation by professionals (based on ED symptomatology and comorbidity, weight changes, interference with social/academic/occupational functioning, etc.). However, as treatment is voluntary, patients can decide to stop the treatment at any time. Patients were also asked to participate in a 6-month follow-up and/or 12-month follow-up. As only 6% (*n* = 14) of the patients participated in one of the follow-up moments, these follow-up data were not used in the present study. No compensation was provided for participation. Data collection started in November 2015 and was finalized in January 2018. A pseudonymized document (only patient codes) was sent to the researchers to ensure that they did not have access to patient names or characteristics. The study was approved by the local ethics board of the psychiatric hospital. The ethics board of the Faculty of Psychology and Educational Sciences of the first author approved retrospective and pseudonymized use of these data (protocol code: G-2018 08 1312 and date of approval 22 August 2018).

### 2.2. Participants

A total of 225 female patients completed questionnaires at admission. From these 225 patients participating at admission, 110 also participated in at least one additional measurement wave, with 43.64% (*n* = 48) participating at admission and during treatment, 16.36% (*n* = 18) participating at admission and at discharge, and 40% (*n* = 44) participating at admission, during treatment, and at discharge. The mean time between Wave 1 and Wave 2 was 11.62 weeks (*SD* = 1.99, range = 5–19.29) and the mean time between Wave 1 and Wave 3 was 19.75 weeks (*SD* = 3.34, range = 11–27.86). Longitudinal analyses in the present study were performed on the data of patients who participated in at least two measurement waves (*n* = 110). Missing data are handled by way of direct likelihood, which means that all data from these 110 subjects are included into the analysis, whether complete or not over the three waves. Given that dropout is shown to depend on observed outcomes, missing completely at random (MCAR) is ruled out, while the corresponding dropout mechanism is still compatible with missing at random (MAR), which applies when dropout depends on observed data but, given these, not further on missing data. Therefore, we opt for methods that are valid under MAR [34]. Table 1 represents the distribution of all the participants over the different measurement waves.

As an unexpectedly high number of patients dropped out during the study (*n* = 115), a cross-tabulation and multivariate analyses of variance (MANOVA) analysis were carried out to compare these patients with the final study sample (*n* = 110) on various ED- and treatment-related variables. This indicated no significant differences on diagnosis (χ^2^_(4)_ = 4.43, *p* = 0.35), age, BMI at admission, illness duration, and amount of previous admissions at the treatment center. Table 2 displays all univariate *F*-values. However, patients who dropped out had a shorter treatment duration (*M* = 10.54 weeks, *SD* = 8.39) than patients in the final study sample (*M* = 21.29 weeks, *SD* = 5.20). Moreover, they differed significantly on identity functioning and ED symptomatology at admission [Wilks’Ʌ = 0.87, *F*(10,214) = 3.22, *p* < 0.01], with patients who dropped out scoring higher on identity synthesis, commitment making, and identification with commitment, and lower on identity confusion and drive for thinness.

In the final study sample (*n* = 110), age ranged from 14 to 45 years (*M* = 20.87, *SD* = 6.08), the mean BMI was 17.27 (*SD* = 4.10, range = 10.35–35.52), and the mean illness duration was 5 years (*SD* = 5.60, range = 0–23). For 68.2% (*n* = 75) of the patients, it was their first admission at the unit. Conversely, 26.4% (*n* = 29) already underwent one or more treatments at the unit, and for 5.5% (*n* = 6) no information was available regarding previous treatments. According to DSM-5 [4], 48.2% of the patients (*n* = 53) met criteria for AN-R, 17.3% (*n* = 19) met criteria for AN-BP; 11.8% (*n* = 13) met criteria for BN; 4.5% (*n* = 5) met criteria for BED, 15.5% (*n* = 17) met criteria for EDNOS, and for 2.7% (*n* = 3) no information was available regarding diagnosis.

### 2.3. Measures

#### 2.3.1. ED Symptomatology

The Eating Disorder Inventory-2 [35] can track progress and outcome in clinical ED populations [36]. The present study focused on the three risk scales that measure some of the central characteristics of an ED. Drive for Thinness (7 items) refers to a preoccupation with dieting and weight and the wish of being thinner. Body Dissatisfaction (9 items) refers to the idea that specific body parts are too large. Bulimia (7 items) refers to uncontrollable overeating and/or self-induced vomiting. All items are scored on a 6-point Likert-type scale (ranging from 1_never to 6_always), with higher mean scores representing more ED symptoms.

#### 2.3.2. Identity Synthesis and Confusion

The Identity Subscale of the Erikson Psychosocial Stage Inventory (EPSI) [37] is a valid measure to assess identity synthesis and identity confusion in adolescents and emerging adults [38]. Both subscales contain six items, scored on a 5-point Likert-type scale (ranging from 1_strongly disagree to 6_strongly agree), with higher mean scores representing more synthesis or confusion.

#### 2.3.3. Identity Processes

The Dimensions of Identity Development Survey [13] is proven to be a valid questionnaire to measure the identity processes in both adolescent and adult samples. All five subscales (exploration in breadth, commitment making, exploration in depth, identification with commitment, and ruminative exploration) consist of five items, scored on a 5-point Likert-type scale (ranging from 1_strongly disagree to 5_strongly agree). Mean scores were calculated for all identity processes.

### 2.4. Statistical Analyses

First, in order to compare certain subgroups of patients on identity functioning, MANOVAs were used with age groups (adolescent ≤18 year/adult >18 year), ED subtypes (restrictive/binge–purging), and previous admissions at the treatment center (yes/no) as independent variables and identity dimensions at admission as dependent variables.

Second, univariate latent growth curve (LGC) modeling within a structural equation modeling framework was used in Mplus (version 7.4) [39] to examine developmental trajectories of identity synthesis and confusion and the identity processes throughout therapy. Again, data from all subjects (*n* = 110) are included into the analysis, whether they completed the study or not, as missing data are handled by way of direct likelihood. This LGC technique models inter-individual variability and change over time by estimating variances and means of the intercept and slope growth factors [40]. The intercept represents the initial level of the outcome variable. In the present study, its factor loadings are fixed to 1. The slope represents the rate of change over time. However, as the intervals of the present study are not equally spaced in time and individuals are not measured at the same time intervals, random slopes are used, allowing each participant to have a different slope (following example 6.12 in the Mplus manual) [39]. Adopting a definition variable approach [41], the factor loadings of the slopes are individually fixed to participants’ time intervals (expressed in weeks) between the measurement waves (i.e., definition variables). More specifically, for Wave 1, slopes are fixed to 0 for all participants. For Wave 2, slopes are estimated based on the number of weeks between Wave 1 and Wave 2 for each participant. For Wave 3, slopes are estimated based on the number of weeks between Wave 1 and Wave 3 for each participant. With regard to model fit, no global fit indices exist in this random model design, as only likelihood-based fit indices are available [41].

Finally, multivariate LGC modeling was used to investigate the simultaneous change of identity functioning and ED symptomatology. Each LGC model examined the development of one identity dimension and one ED symptom. This approach allows for an examination of the extent to which the development of identity and ED symptoms are interrelated throughout therapy [42].

All Cronbach’s alpha coefficients are presented in Table S1 and ranged from 0.71 to 0.96.

## 3. Results

### 3.1. Identity Functioning as a Function of Age Groups, ED Subtypes and Admission History

In the longitudinal sample (*n* = 110), the MANOVAs indicated no significant differences on identity functioning at admission between adolescent (≤18 year) and adult (>18 year) patients (Wilks’Ʌ = 0.90, *F* (7,102) = 1.66, *p* = 0.13), patients with restrictive versus bingeing–purging ED subtypes (Wilks’Ʌ = 0.92, *F* (7,99) = 1.29, *p* = 0.27), and patients with and without previous admissions at the treatment center (Wilks’Ʌ = 0.89, *F* (7,96) = 1.77, *p* = 0.10). Table S2 displays the within-time Pearson correlations of identity functioning with ED symptomatology at times 1–3 (*n* = 110).

### 3.2. Identity Functioning throughout Treatment

Table 3 presents the seven univariate latent trajectories of identity functioning in the patient sample throughout treatment (*n* = 110). Patients significantly decreased on identity confusion and significantly increased on identity synthesis and all adaptive identity processes (commitment making, identification with commitment, exploration in breadth, exploration in depth) over time.

### 3.3. Linking Identity Changes to Changes in ED Symptomatology

All multivariate LGC models with identity functioning and ED symptoms indicated that patients experienced a significant decrease of ED symptomatology (drive for thinness, body dissatisfaction, bulimia) throughout treatment (*n* = 110). With regard to drive for thinness, the intercepts ranged from 5.02 to 5.04, and the slopes were consistently −0.03 (*p* < 0.001) (Table S3). With regard to body dissatisfaction, intercepts ranged from 5.02 to 5.04 and slopes were consistently −0.06 (*p* < 0.001) (Table S4). With regard to bulimia, intercepts ranged from 2.34 to 2.36, and slopes were consistently −0.03 (*p* < 0.001) (Table S5). Table 4 gives an overview of the covariations between the intercepts and slopes of the identity measures and the ED symptoms. Intercepts of drive for thinness and body dissatisfaction were positively related to intercepts of ruminative exploration, whereas they were negatively related to intercepts of identity synthesis and identification with commitment. This means that patients with higher scores on drive for thinness and body dissatisfaction at admission, also presented with worse identity functioning. Similarly, slope factors of drive for thinness and body dissatisfaction were positively related to slope factors of identity confusion and ruminative exploration, meaning that increases (or decreases) in these ED symptoms were related to increases (or decreases) in these maladaptive identity measures. Correspondingly, ED symptoms were negatively related to slope factors of identity synthesis and identification with commitment, meaning that increases (or decreases) in these ED symptoms were related to decreases (or increases) in these adaptive identity measures.

## 4. Discussion

Identity development is a growing area of interest within the field of eating disorders (EDs). Previous research in clinical samples already established that patients with an ED encounter substantial identity problems [7,9,43], and that also, in community samples, a close relation has been found between ED symptomatology and identity confusion, e.g., [1,2,15,44]. Although their longitudinal association has already been investigated in community samples, no study has addressed this topic in an inpatient ED sample. The present three-wave longitudinal design enabled us to investigate identity functioning throughout an ED treatment (at admission, during treatment, and at discharge) and to explore its interrelation with ED symptomatology over time.

Contrary to our expectations, we did not find significant differences on identity functioning at admission between adolescent and adult patients, patients with restrictive versus bingeing–purging ED subtypes, and patients with and without previous admissions at the treatment center. It seems that all inpatients regardless of their age, ED subtype or treatment history experience identity-related issues somewhat to the same extent at intake. It can be that they all feel bad due to their ED, and thus all report identity-related issues at admission. This finding again emphasizes the need to focus on identity issues in the population of patients with ED at large, as previously highlighted by Stein [43], Amianto [45], and Oldershaw [46].

Throughout the ED treatment, patients’ identity functioning improved significantly, as shown by increases in identity synthesis, commitment processes, and adaptive exploration processes. The treatment context appears to stimulate patients in exploring identity alternatives and comparing such alternatives with internal standards. Additionally, patients improved in committing to life decisions and identifying with them. Finally, they experienced a stronger sense of identity, with less fragmentation and confusion about themselves [6]. Additionally, patients also experienced a significant decrease in ED symptomatology (drive for thinness, body dissatisfaction, bulimia) throughout treatment.

When relating the identity changes to the changes in ED symptomatology, interesting findings occurred. In line with previous research in community adolescents [2], changes in identity synthesis and confusion were related to changes in drive for thinness and body dissatisfaction. More specifically, patients who showed decreasing scores on drive for thinness and body dissatisfaction, also showed decreasing scores on identity confusion and increasing scores on identity synthesis. Similarly, these patients also showed decreasing scores on ruminative exploration and increasing scores in identification with commitment. It is important to note that these associations only focus on simultaneous developments and no conclusions can be made on directionality of effects. Our findings also confirm earlier qualitative studies, in which the recovery from an ED is described as a complex and challenging expedition, which involves letting go of the ED-identity and discovering a non-ED-centric identity [47,48,49]. When they experience a coherent and stable sense of identity, patients with an ED may be better equipped to successfully manage stressful situations which are inherent in recovery, such as weight gain, and challenging body image concerns [50]. These findings also are in line with the Maudsley model of anorexia nervosa treatment for adolescents and young adults (MANTRa) which stresses the importance of identity work in patients with EDs, certainly in adolescents [51]. In MANTRa, role models are sought to construct non-illness-driven alternative values and beliefs and enhance identity exploration and formation [51,52]. Further, we also found significant associations between the intercepts of the identity dimensions and ED symptoms. This result indicates that patients with the highest levels of drive for thinness and body dissatisfaction at admission also presented with the worst identity functioning—highlighting the importance of screening for identity functioning at admission and including identity work in the treatment of patients with an ED.

Surprisingly, almost no significant associations were found between identity changes and changes in bulimic symptoms. A possible explanation for this result might be related to the ED treatment context, in which patients are carefully observed by staff and other patients. Such a treatment context is characterized by close monitoring and behavioral management—such as well-planned and structured eating moments in group, shared toilets, laboratory evaluation, and early discharge as a result of breaking admission agreements—all aiming to limit binge eating/purging immediately at admission [53,54]. Hence, it is possible that early decreases in bulimic symptoms are not intrinsically motivated or related to self-related changes, but are especially caused by contextual factors. Hence, it would be interesting to investigate changes in bulimic symptoms and identity in an outpatient context as well.

Finally, at admission, patients who dropped out during the study (*n* = 115) presented with higher scores on identity synthesis and the commitment processes and lower scores on identity confusion than patients in the final study sample (*n* = 110). As these patients had a shorter treatment duration, it is possible that identity synthesis, confusion, and (identification with) commitment are important individual characteristics that could differentiate patients who respond more quickly to ED therapy, and hence, are discharged sooner. However, it is important to bear in mind that we do not have any information on the reason of discharge in the present study. Future studies with more patients at each of the different measurement waves are urgently needed, to (dis)confirm the findings of the present study.

While this study has provided important insights into identity functioning in an ED treatment context, some additional limitations must be considered. First, due to the treatment setting, all participants were female and results cannot be generalized for the entire ED population. Previous research has found differences in identity functioning between community men and women [55,56], which may also exist in an ED context. Second, we did not have information on the reasons of discharge (e.g., successful treatment, patient’s decision, rule-breaking behavior) or reasons of inpatient drop-out (e.g., time or psychological burden). It would be interesting to include this information in future research as it could further strengthen our conclusions and possibly correct assumptions that were made in the present study. In addition, the second measurement wave of the present study was planned after 12 weeks of treatment, but the actual time range was rather large (5–19.29 weeks). Although it is difficult to implement a strict study plan in a clinical setting, a smaller time range between the measurement waves can help in streamlining the results and conclusions. Fourth, the present study focused on the simultaneous development of identity and ED symptomatology throughout treatment, but did not investigate the directionality of effect. While previous research in community samples indicated a bidirectional relationship [2], cross-lagged analyses in future clinical research may replicate these findings in a clinical context. Finally, only self-report questionnaires were used, which may increase reporting bias and inflated correlations between studied variables. Including other methods (e.g., interviews or reports by family members) are advised in future research.

## 5. Conclusions

The present study is the first comprehensive investigation of personal identity development at multiple levels in an ED treatment context. When relating identity changes to the changes in ED symptomatology, interesting findings occurred. Patients who showed decreasing scores on drive for thinness and body dissatisfaction also showed decreasing scores on identity confusion and increasing scores on identity synthesis. In the same vein, these patients also showed decreasing scores on ruminative exploration and increasing scores in identification with commitment. Based on these findings, bolstering identity functioning (e.g., identifying with life commitments, breaking the ruminative identity cycle) and treating body size/weight concerns in therapy could be helpful to decrease ED symptoms.

## Figures and Tables

**Table 1:** Distribution of the participants (*n* = 225) over the different measurement waves.

	Measurement Wave 1 at Admission	Measurement Wave 2 during Treatment	Measurement Wave 3 at Discharge	Number of Participants		
Wave 1 ^a^	X			115		
Wave 1 + Wave 2	X	X		48	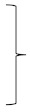	
Wave 1 + Wave 3	X		X	18		*n* = 110
Wave 1 + Wave 2 + Wave 3	X	X	X	44		

^a^ Each of the different rows in this table include different patients, e.g., patients who belong to group [Wave 1 + Wave 2] are different patients than those who belong to group [Wave 1 + Wave 2 + Wave 3] etc. At Wave 1, 225 (115 + 48 + 18 + 44) patients participated in the study; at Wave 2, 92 (48 + 44) patients participated, and at Wave 3, 62 (18 + 44) patients participated.

**Table 2:** (M)ANOVAs for mean differences on descriptive and study variables at admission between drop out sample (*n* = 115) and final study sample (*n* = 110).

	Drop Out Sample (*n* = 115)	Final Study Sample (*n* = 110)		
Variable	*M* (*SD*)	*M* (*SD*)	*F*-Value	Partial η^2^
Age	21.50 (6.42)	20.87 (6.08)	0.57	0.00
BMI at admission	18.16 (5.01)	17.27 (4.10)	2.13	0.01
Illness duration (years)	5.78 (5.70)	5.00 (5.57)	1.03	0.31
Treatment duration (weeks)	10.54 (8.39)	21.29 (5.20)	106.77 ***	0.38
Identity dimensions				
Identity synthesis	3.09 (0.64)	2.73 (0.78)	14.62 ***	0.06
Identity confusion	2.56 (0.68)	3.39 (0.68)	7.56 **	0.03
Commitment making	3.49 (0.83)	3.10 (1.02)	10.04 **	0.04
Identification with commitment	3.40 (0.75)	2.93 (0.82)	7.09 **	0.03
Exploration in breadth	3.59 (0.65)	3.61 (0.74)	3.31	0.02
Exploration in depth	3.30 (0.73)	3.39 (0.77)	1.50	0.07
Ruminative exploration	2.67 (0.87)	3.58 (0.95)	0.29	0.00
Eating disorder symptomatology				
Drive for thinness	4.80 (1.08)	5.08 (0.88)	4.57 *	0.02
Body dissatisfaction	4.95 (0.99)	5.02 (0.90)	0.34	0.00
Bulimia	2.77 (1.38)	2.47 (1.33)	2.72	0.01

*M* = Mean; *SD* = standard deviation; Partial η^2^ = partial eta squared. All identity variables have a possible range of 1–5. All ED variables have a possible range of 1–6. * *p* < 0.05. ** *p* < 0.01. *** *p* < 0.001.

**Table 3:** Parameter estimates of univariate latent growth curve models on identity synthesis, confusion, and processes (*n* = 110).

	Intercept		Slope	
Variable	*M*	∆	*M*	∆
Identity synthesis	2.712 ***	0.442 ***	0.025 ***	0.001
Identity confusion	3.529 ***	0.377 ***	−0.015 ***	0.000
Commitment making	2.920 ***	0.659 ***	0.017 **	0.000
Identification with commitment	2.764 ***	0.625 ***	0.010 *	0.001
Exploration in breadth	3.527 ***	0.269 *	0.013 **	0.000
Exploration in depth	3.334 ***	0.361 ***	0.013 ***	0.000
Ruminative exploration	3.635 ***	0.613 ***	−0.008	0.001

*M* = mean; ∆ = variance. Mean slopes represent the mean identity change for every week in treatment. * *p* < 0.05. ** *p* < 0.01. *** *p* < 0.001.

**Table 4:** Covariations between growth factors of ED symptoms and identity functioning derived from multivariate models (*n* = 110).

	Drive for Thinness		Body Dissatisfaction		Bulimia	
Variable	Intercept	Slope	Intercept	Slope	Intercept	Slope
Identity synthesis						
Intercept	−0.214 *	0.003	−0.270 **	0.001	0.014	0.000
Slope	0.003	−0.001 **	0.005	−0.001 *	−0.002	0.000
Identity confusion						
Intercept	0.050	0.001	0.106	0.003	0.018	−0.001
Slope	0.001	0.001 *	0.000	0.001 *	0.002	0.000
Commitment making						
Intercept	−0.109	−0.004	−0.096	−0.011	0.117	−0.004
Slope	0.007	−0.001	0.004	0.000	−0.003	0.000
Identification with commitment						
Intercept	−0.172 *	−0.003	−0.198 *	−0.005	0.065	−0.002
Slope	0.004	−0.001 ˟	0.006	−0.001 *	−0.002	0.000
Exploration in breadth						
Intercept	−0.064	0.003	−0.030	−0.001	0.165 ˟	−0.002
Slope	0.001	0.000	0.000	0.000	−0.004	0.000
Exploration in depth						
Intercept	−0.046	0.003	−0.145 *	0.003	0.024	0.000
Slope	0.000	−0.001 *	0.002	0.000	−0.005	0.000
Ruminative exploration						
Intercept	0.226 *	−0.002	0.276 **	0.001	0.119	−0.002
Slope	−0.001	0.001 ^†^	−0.005	0.001 *	−0.005	0.000

^†^ *p* = 0.06. ˟ *p* = 0.05. * *p* < 0.05. ** *p* < 0.01.

## Data Availability

The data that support the findings of this study are available from the corresponding author upon reasonable request (laurence.claes@kuleuven.be). The data are not publicly available due to privacy restrictions of the research participants.

## Supplementary Materials

The following supporting information can be downloaded at: https://www.mdpi.com/article/10.3390/nu16050591/s1, Table S1: Cronbach’s alpha coefficients of the study variables; Table S2: Within-time Pearson correlations of identity functioning with ED Symptomatology at times 1–3 (*n* = 110); Table S3: Parameter estimates of seven multivariate latent growth curve models with drive for thinness (*n* = 110); Table S4: Parameter estimates of seven multivariate latent growth curve models with body dissatisfaction (*n* = 110); Table S5: Parameter estimates of seven multivariate latent growth curve models with bulimia (*n* = 110).
