# Supplementary material for: Identity Functioning in Patients with an Eating Disorder: Developmental Trajectories throughout Treatment
Source: Nutrients. 2024 Feb 21;16(5):591. doi: 10.3390/nu16050591 (PMC10935138; doi:10.3390/nu16050591)
Supplement: Supplementary file 1 [file nutrients-16-00591-s001.zip › nutrients-2854005-supplementary.pdf]

Supplementary Materials

Table S1. Cronbach's Alpha Coefficients of the Study Variables

	Cronbach's alpha		
	Wave 1	Wave 2	Wave 3
Variable	*n*=110	*n*=92	*n*=62
**EDI-2**			
Drive for thinness	.87	.88	.93
Body dissatisfaction	.88	.88	.93
Bulimia	.94	.92	.86
**EPSI**			
Identity synthesis	.76	.80	.85
Identity confusion	.71	.80	.76
**DIDS**			
Commitment making	.96	.94	.95
Identification with commitment	.85	.90	.90
Exploration in breadth	.82	.82	.85
Exploration in depth	.89	.74	.75
Ruminative exploration	.90	.88	.90

Table S2. Within-Time Pearson Correlations of Identity Functioning with ED symptomatology at Times 1-3 (n=110)

Variable	Drive for thinness	Body dissatisfaction	Bulimia
Identity synthesis			
Time 1	**-.44****	**-.58****	-.10
Time 2	**-.58****	**-.55****	-.14
Time 3	**-45****	**-.54****	-.17
Identity confusion			
Time 1	.11	**.31***	-.03
Time 2	**.41****	**.37***	.21
Time 3	**.33***	**.44****	.27
Commitment making			
Time 1	-.21	**-.33***	.02
Time 2	-.23	-.09	-.08
Time 3	-.15	-.26	-.15
Identification with commitment			
Time 1	-.28	**-.44****	-.03
Time 2	**-.34***	**-.35***	-.05
Time 3	-.26	**-.39****	-.20
Exploration in breadth			
Time 1	-.06	-.10	.14
Time 2	-.18	-.05	.26
Time 3	.01	-.06	.26
Exploration in depth			
Time 1	.00	-.13	.02
Time 2	-.07	-.17	.04
Time 3	-.23	-.13	-.07
Ruminative exploration			
Time 1	**.32***	**.47****	.08
Time 2	**.45****	**.40****	.28
Time 3	**.41****	**.56****	.28

*p<.05, **p<.01

Table S3. Parameter Estimates of 7 Multivariate Latent Growth Curve Models with Drive for Thinness (n=110)

	Intercept		Slope	
Variable	*M*	Δ	*M*	Δ
Identity synthesis	2.702***	0.426***	0.027***	0.001
Drive for thinness	5.036***	0.537*	-0.061***	0.002
Identity confusion	3.533***	0.379***	-0.017***	0.000
Drive for thinness	5.029***	0.530*	-0.061***	0.002
Commitment making	2.906***	0.683***	0.019**	0.001
Drive for thinness	5.035***	0.530*	-0.062***	0.002
Identification with commitment	2.753***	0.580***	0.011*	0.001
Drive for thinness	5.034***	0.532*	-0.061***	0.002
Exploration in breadth	3.524***	0.265*	0.013**	0.000
Drive for thinness	5.027***	0.496*	-0.061***	0.002
Exploration in depth	3.327***	0.376***	0.014***	0.000
Drive for thinness	5.032***	0.524*	-0.061***	0.002
Ruminative exploration	3.646***	0.602***	-0.010*	0.001
Drive for thinness	5.022***	0.495*	-0.060***	0.002

Note. M = Mean; Δ = Variance. Mean slopes represent the mean identity change for every week in treatment.
*p<.05. **p<.01. ***p<.001.

Table S4. Parameter Estimates of 7 Multivariate Latent Growth Curve Models with Body Dissatisfaction (n=110)

	Intercept		Slope	
Variable	*M*	Δ	*M*	Δ
Identity synthesis	2.714***	0.438***	0.025***	0.001
Body dissatisfaction	5.044***	0.551**	-0.028**	0.001
Identity confusion	3.529***	0.385***	-0.015***	0.000
Body dissatisfaction	5.039***	0.531**	-0.027***	0.001
Commitment making	2.922***	0.675***	0.017**	0.000
Body dissatisfaction	5.044***	0.502**	-0.026***	0.000
Identification with commitment	2.761***	0.598***	0.010*	0.001
Body dissatisfaction	5.039***	0.536**	-0.027***	0.001
Exploration in breadth	3.527***	0.266*	0.013**	0.000
Body dissatisfaction	5.047***	0.469**	-0.027***	0.000
Exploration in depth	3.334***	0.361***	0.013***	0.000
Body dissatisfaction	5.045***	0.513**	-0.027***	0.001
Ruminative exploration	3.632***	0.667***	-0.008	0.001*
Body dissatisfaction	5.035***	0.571**	-0.027***	0.001

Note. M = Mean; Δ = Variance. Mean slopes represent the mean identity change for every week in treatment.
**p*<.05. ***p*<.01. ****p*<.001.

Table S5. Parameter Estimates of 7 Multivariate Latent Growth Curve Models with Bulimia (n=110)

	Intercept		Slope	
Variable	*M*	Δ	*M*	Δ
Identity synthesis	2.708***	0.438***	0.026***	0.001
Bulimia	2.352***	1.355***	-0.032***	0.001
Identity confusion	3.530***	0.380***	-0.016***	0.000
Bulimia	2.344***	1.360***	-0.032***	0.001
Commitment making	2.914***	0.674***	0.018**	0.001
Bulimia	2.349***	1.361***	-0.032***	0.001
Identification with commitment	2.763***	0.628***	0.010*	0.001
Bulimia	2.356***	1.371***	-0.032***	0.001
Exploration in breadth	3.531***	0.297*	0.013**	0.000
Bulimia	2.340***	1.369***	-0.031***	0.001
Exploration in depth	3.331***	0.374***	0.013***	0.000
Bulimia	2.350***	1.348***	-0.032***	0.001
Ruminative exploration	3.639***	0.611***	-0.009	0.001
Bulimia	2.348***	1.370***	-0.032***	0.001

Note. M = Mean; Δ = Variance. Mean slopes represent the mean identity change for every week in treatment.
**p*<.05. ***p*<.01. ****p*<.001
